# Multiple lung metastases from typical type A thymoma after long-term follow-up: a case report

**DOI:** 10.1186/s13019-026-04045-5

**Published:** 2026-04-16

**Authors:** Keita Tobari, Tsuyoshi Takahashi, Atsushi Sano

**Affiliations:** Department of Thoracic Surgery, Toho University Sakura Medical Center, 564-1 Shimoshizu, Sakura, 285-8741 Chiba Japan

**Keywords:** Type A thymoma, Pulmonary metastasis, Thymoma recurrence, Slow-growing tumor, Prostate cancer, Secondary malignancy, Mediastinal tumor, Long-term follow-up

## Abstract

**Background:**

Typical type A thymomas are slow-growing tumors generally associated with a favorable clinical course, and distant metastasis is exceedingly rare.

**Case presentation:**

We report a case of multiple lung metastases from a typical type A thymoma in a 74-year-old man, identified 9 years after the initial detection of an anterior mediastinal nodule. The patient underwent simultaneous thymectomy and wedge resection of the pulmonary nodule. Histopathological examination confirmed a diagnosis of typical type A thymoma with pulmonary metastases. Postoperatively, the patient was also diagnosed with prostate cancer as a secondary malignancy. At 2-year follow-up, without any postoperative treatment, there was no evidence of progression or development of new pulmonary lesions.

**Conclusion:**

This case underscores the potential for distant metastasis in patients with otherwise indolent, typical type A thymomas, highlighting the need for long-term surveillance.

## Background

Type A thymomas, especially typical type A thymomas, are generally considered slow-growing tumors associated with a favorable clinical course, and distant metastasis is exceedingly rare. We report a case of rare and extremely slow-growing multiple lung metastases from a typical type A thymoma.

## Case presentation

**Fig. 1:**
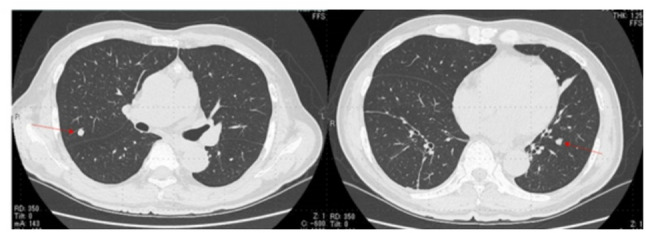
Multiple pulmonary nodules without spiculation are observed. A total of 14 similar nodules is present in both lungs, with a maximum diameter of 9 mm

**Fig. 2:**
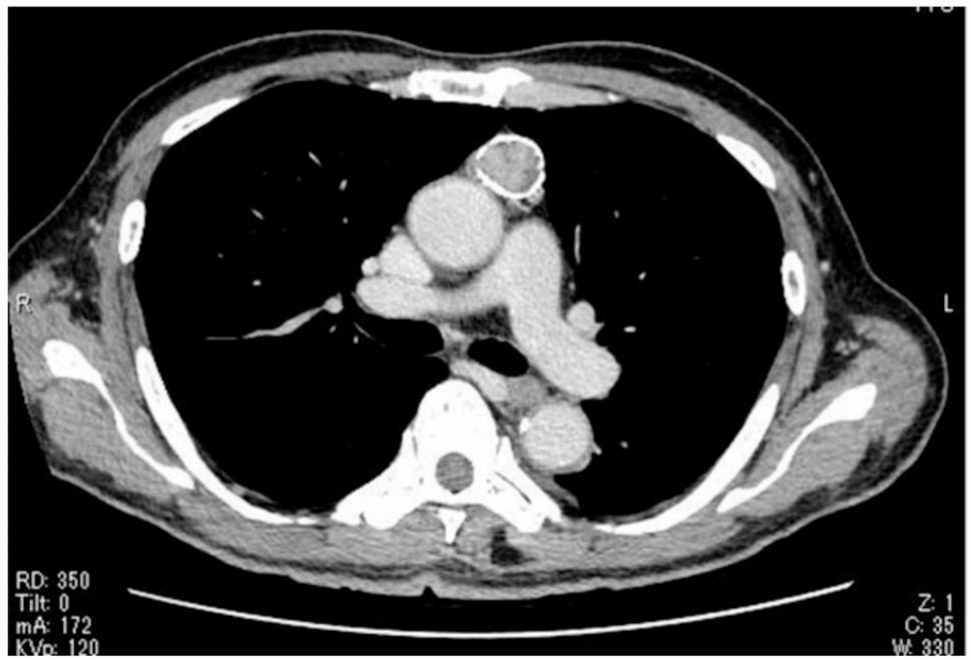
Chest computed tomography shows a 22-mm anterior mediastinal tumor with ring-shaped calcification

**Fig. 3:**
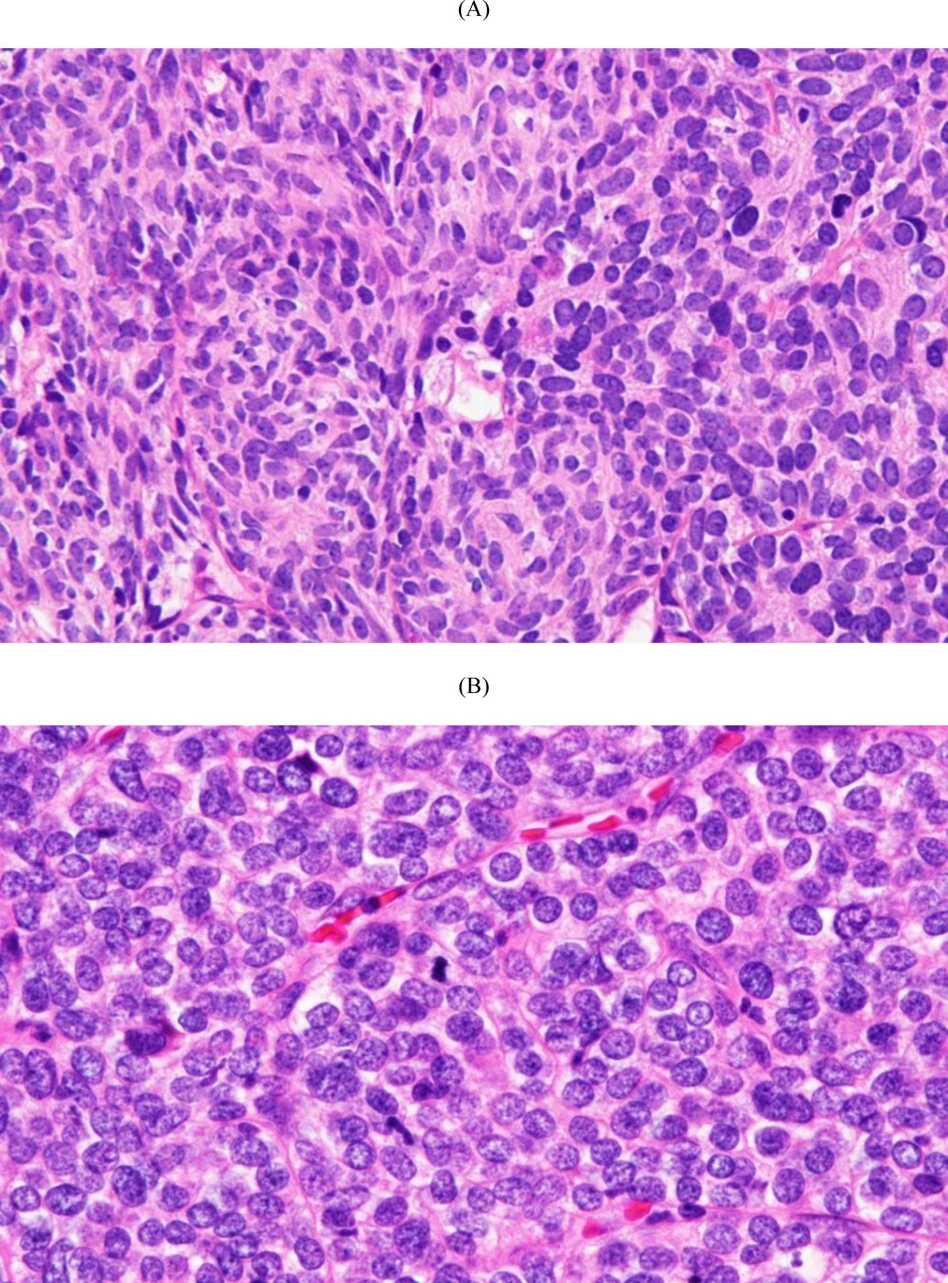
(**A**) Spindle-shaped tumor cells proliferating in the thymus without cytologic atypia; the pathologic diagnosis is typical type A thymoma (hematoxylin and eosin staining, original magnification ×400) (**B**) Pulmonary tumor specimen showing metastatic typical type A thymoma (hematoxylin and eosin staining, original magnification ×400)

A 74-year-old man presented to our hospital for computed tomography (CT) evaluation following pyelonephritis. Chest CT revealed numerous small nodules (maximum diameter: 9 mm) in both lungs (Fig. 1), along with a 22-mm anterior mediastinal nodule exhibiting ring-shaped calcification (Fig. 2). The mediastinal nodule had been noted on a CT 9 years earlier, at which time no lung nodules were present. Blood tests showed an elevated prostate-specific antigen level of 7.1 ng/mL (normal: ≤4.0 ng/mL), with no elevation of other tumor markers and no evidence of anti-acetylcholine receptor antibodies. The lung nodules were suspected to be metastases originating from the mediastinum or prostate cancer. The anterior mediastinal nodule was suspected to be a thymoma. The patient underwent resection of the anterior mediastinal tumor and wedge resections of the right upper lobe pulmonary nodules via median sternotomy for diagnostic and therapeutic purposes. Pathological examination of the mediastinal tumor confirmed a typical type A thymoma (Fig. 3A) with pulmonary metastases (Fig. 3B). The TNM classification was pT1N0M1b, stage Ⅳb, and the Masaoka staging system also classified it as stage Ⅳb. The patient was diagnosed with prostate cancer postoperatively. Given the favorable prognosis associated with typical type A thymoma, the patient managed with observation without adjuvant therapy. Over the 2 years following surgery, no new nodules developed, and the size of the existing nodules remained stable.

## Discussion and conclusions

The patient developed multiple lung metastases from a typical type A thymoma and was also diagnosed with prostate cancer as a second primary malignancy during the 9 years between the initial chest CT finding of the anterior mediastinal tumor and surgery. However, the timeline of pulmonary metastasis development from thymoma during those 9 years remains unclear. Specifically, it is unknown whether the metastasis occurred before or after the onset of prostate cancer. Given the absence of growth and new pulmonary metastases during the 2 years following surgery, we speculate that the pulmonary metastasis may have developed prior to the prostate cancer.

An atypical type A thymoma, classified as a subtype by the World Health Organization classified in 2015, is a newer variant with a greater tendency for invasion and a higher risk of metastasis and recurrence compared to the typical type A thymoma [1, 2]. In addition to the usual features of type A thymomas, the pathological picture includes comedo-type tumor necrosis, increased mitotic activity (> 4 mitoses per 2 mm^2^), and nuclear crowding, any or all of which are considered highly malignant [3]. Atypical type A thymoma components can be found mixed with type AB thymomas, and careful attention is warranted even when the pathological diagnosis is more malignant than type AB [4, 5]. Type A thymomas, especially typical type A thymomas such as the one in our case, are generally considered slow-growing tumors associated with a favorable clinical course, and distant metastasis is exceedingly rare. Tatematsu et al. reported a rare case in which a typical type A thymoma and a solitary pulmonary metastasis were identified simultaneously [6]. No additional therapy was administered, and the patient has been observed without evidence of recurrence during follow-up.

Systemic therapy is the treatment of choice for patients with metastatic or unresectable thymomas. Although there is no established treatment consensus for patients with advanced thymoma, the recommended initial treatment includes cyclophosphamide, doxorubicin, and cisplatin rather than other systemic therapies [7]. Murakawa et al. [8] reported that in patients with stage IVa thymoma, localized control and improved prognosis can be achieved through cytoreduction with resection of visible disseminated nodules or extrapleural pneumonectomy combined with radiation therapy. These findings suggest that surgical intervention may benefit patients with non-curative thymomas. In our case, the patient received no postoperative therapy; however, he remained free of local recurrence or progression of thymoma or metastasis.

Engels et al. [9] reported an increased risk of developing secondary cancers in patients with thymomas. In their study, 9% of patients with thymoma subsequently developed a second malignancy. The most common secondary cancers were gastrointestinal cancers (27.8%; standardized incidence ratio [SIR]: 1.8), followed by respiratory cancers (17.8%; SIR: 1.5), prostate cancer (15.5%; SIR: 1.3), non-Hodgkin lymphoma (11%; SIR: 4.7), and breast cancer (6.7%; SIR: 1.2). In our case, the patient developed prostate cancer during the 9-year interval between the first chest CT evaluation and surgery. The exact mechanisms of secondary carcinogenesis related to thymoma remain unconfirmed due to the limited number of reported cases. Nonetheless, it may be associated with impaired T-cell immune function in the thymus caused by the tumor or its treatment [9].

Postoperative surveillance of thymoma is recommended for at least 5 years, in accordance with guidelines for other solid organ malignancies. In this case, the patient had a typical type A thymoma and was diagnosed with prostate cancer 9 years after an incidental finding of an anterior mediastinal thymoma on chest CT. We believe that an effective strategy is to follow up patients with thymoma for at least 10 years, regardless of histological type or disease stage.

In conclusion, we report a case of rare and extremely slow-growing multiple lung metastases from a typical type A thymoma.

## Data Availability

Not applicable.

## Abbreviations

CT: Computed tomography
SIR: Standardized incidence ratio
